# Observations on Gun-Shot Wounds

**Published:** 1842-01-01

**Authors:** W. Poyntell Johnston


We welcome to our pages the following remarks, and the accompanying case from our late co-editor,—the first of a series of three or more—not merely for the value of the articles themselves, but for the example which they furnish of amplitude without prolixity,—excellencies among the greatest desiderata in the art of clinical reporting.

### *Observations on Gun-Shot Wounds.* By W. Poyntell Johnston, M. D.

A case of gun shot wound, involving the question whether an amputation at or near the shoulder joint was necessary to afford the patient a hope of recovery, presented itself to me three weeks since. The details of this interesting case I hope, through the kindness of the attending physicians, to offer to you when the treatment shall have terminated. It was the unanimous opinion of all consulted that immediate amputation was required. The patient acceded to the necessity of the operation, but refused to submit to it, preferring the risk of almost certain death. From present appearances he may possibly recover, without the loss of the limb.

I refer to this case simply to call attention to the great uncertainty of prognosis in all cases of serious gun shot wounds, even where the question of amputation of a limb is not involved. In looking over my case book I find notes of several cases of this kind, in which the estimate of danger to life, formed at the moment of the accident, was not at all justified by the result. From these I select two of opposite character; one followed by entire recovery, where but little hope could be entertained from the nature of the wound; the other, by death, although a favourable result was confidently anticipated. (In both instances I refer to the prognosis formed at the moment of the accident.) If these cases present sufficient interest, as introductory to the one alluded to of later date, they are at your disposal. I would not offer them, however, unless each presented other points of interest of greater value than the mere proof that prognosis in gun shot wounds is uncertain.

### Case I.—*Gun-shot Wound. Small shot traversing arm, entering side, &c.*

Margaret Baugh, domestic, æt. 14, large, and very strongly built, and of good constitution; has never been seriously sick; was engaged, between five and six o'clock in the morning, in arranging the breakfast table at a farm house in Richmond, about four miles from Philadelphia, when a young lad, while playing in the same room with a gun charged with small shot, threatened to shoot her. He was standing with the gun resting in the bend of his left arm, and pointed towards Margaret in a horizontal direction, when it was accidentally discharged. The patient fell upon a chair, where she was found immediately afterwards by the members of the family, screaming with pain, and bleeding profusely from the arm and side. A physician saw her in half an hour and dressed the wounds; after which she was carefully transported to the city in a covered wagon.

Visited at half past eleven, five hours after the accident; she is found in the following condition: She is very pale and feeble; her pulse scarcely perceptible, and her extremities cold. She has lost, according to her mother's account, three pints of blood from her wounds, and had vomited blood during the whole of her journey to the city. The dressings around the arm are saturated with blood, while those on the side are merely discoloured. On removing these, there are seen on the external surface of the right arm, between the insertion of the deltoid muscle and the articulation of the elbow, thirty orifices. Most of these are small, round, and smooth. A few are oblong and jagged. These correspond with the entrance of the shot; the great mass occupies about the middle of the space above mentioned; two or three reach as high as the insertion of the deltoid, and three or four have apparently entered the articulation at the elbow. On the inside of the arm exist twelve irregular orifices, caused by the escape of as many shot, which have traversed the biceps from side to side, and from before backwards, traversing the course of the brachial artery. Blood still oozes from a few of these openings, while the remainder are filled with small black coagula. The arm is neither red nor tumefied, and the pain is very slight. Finally, the right side of the thorax presents twenty similar orifices disposed as follows: One is situated at the inferior and lateral part of the right mamma, which is remarkably developed for the age of the patient; three others, a little lower, and placed very nearly on the same horizontal plane, correspond with the inferior lobe of the right lung; four others occupy a situation corresponding with the distance separating the lung from the liver; and the remaining twelve, disposed in a mass, and occupying a space about half the size of the hand, are opposite the convex portion of the liver. The skin, in the vicinity of the wounds, is pale and cold. The girl complains of pain in the side, which she attributes to the laceration of the skin by the shot. The pulsations of the radial artery *cannot be felt* on the injured side, but the pulse in the opposite member is distinctly perceptible, although feeble.

The prognosis in this case was exceedingly grave. There existed probably an injury of the brachial artery, inferrible from the direction of so many shot all traversing its course within so small a space—from the amount of hæmorrhage from such small orifices, (three pints in five hours,) notwithstanding the coagula which blocked up the openings,—from the entire loss of pulsation at the wrist, without tumefaction, and consequently without arrest of the circulation by compression, and without any evidence of local stupor—either palor, puffiness, or loss of sensibility.

But the injury of the brachial artery—clearly to be inferred—constituted a small item in the sum of dangers. Three shot had evidently penetrated the parenchyma of the lungs, and, of course, traversed the pleura; twelve had entered the substance of the liver, involving the peritoneum; and four had, in all probability, traversed the ascending diaphragm, involving not only the muscle, but both its serous coats: hence inflammation of two serous tissues, and two parenchymata. Added to all this was the vomiting—not expectoration—rendering not impossible injury to the stomach. Directed cold poultice, to be continually moistened with cold water, applied to the arm; simple cerate, followed by poultices, to the side; sinapisms to the stomach and left wrist; hot bricks to the feet.

Aug. 25th. The patient vomited a little greenish matter yesterday, after her wounds were dressed. To day the pulse is fuller, although she is still feeble. Ordered cold lemonade for drink. In the evening V. S. ℥xii.

Aug. 26th. Suffers little pain, except in the axilla and in a line from thence under the mamma. Left cheek flushed. Pulse full, 150. Respiration 45, slightly laborious, but causing no pain. The edges of the wounds offer a whitish colour, and each is surrounded by a marked red areola, about a line in breadth. The intermediate skin offers, in some places, a slight yellowish tinge. In the evening sharp pain in right side. 50 leeches, loco doloris.

Aug. 27th. Slept well. Face pale. Respiration slightly laborious, accelerated. Pulse 135. The pain in the side is completely relieved. The sloughs have come away from several of the orifices. On raising her up to apply the poultice to her side, four other orifices were discovered at the posterior lateral part of thorax; the most superior being on a level with the inferior angle of the scapula. The three others are below the insertion of the diaphragm. On the back, the skin has been torn away by two shot which did not enter the flesh. The cuts are horizontal, and about two inches in length. They are covered by a very thin slough, and moistened by a slight serous discharge. Poultices to side and arm. Lemonade.

Aug. 28th. Drowsy. Does not sleep at night, but doses during the day. Countenance more natural, expressive of anxiety. Pulse moderately full, 130. Respiration, 35, apparently difficult. No pain. The sloughs have nearly all come away from the orifice in the arm, leaving depressions filled with red granulations. The arm does not appear sensibly tumefied, but the skin around the sores is tense. The orifices in the side are still filled by a black slough, and a serous discharge exudes from them. The pain has entirely ceased. On the back, the gutters, caused by the shot ploughing up the skin, are also filled with a dark grayish slough; their direction, instead of being perfectly horizontal, is backwards, and very slightly downwards. She has taken laudanum, forty drops at night, and the effervescing draught during the day. Dressings continued.

Aug. 29. Restless during the night; did not sleep at all. This morning, moans and complains again of pain in the side. Pulse full, quick. Respiration accelerated, and slightly laborious; great heaving of chest at each inspiration.

Aug. 30. Face slightly flushed. Very restless during the night, and did not sleep at all. Pulse full, quick. Respiration more natural than yesterday. The sloughs have all come from the wounds in the side, which offer precisely the same appearances as those of the arm, with the exception of being less deep. The skin surrounding them is indurated. The patient complains of no pain, but moans continually. The same prescription and dressings continued.

Aug. 31. Slept well. Face natural. Pulse moderately full, 120. Respiration, 31, easy and more natural. Says that she suffers no pain in any part. Had a short, dry cough during the night. No expectoration. Commenced taking, yesterday, Antim. Tart., gr. i.; Tinct. Opii ʒi.; Aqua Menth. et Aqua *aa.* ℥iii. A table-spoonful to be taken every two hours. Slight nausea after first and second doses, which has now disappeared. The same prescription and dressings continued. Chicken water and bread for diet. Effervescing draught during the day.

Sept. 1. *Had a slight chill* yesterday about noon, not very marked, which continued for three or four hours, and was followed by profuse perspiration. To-day the countenance is natural. Pulse 150, full. Respiration rather laboured. No pain. The wounds of the arm vary in appearance; the greater number are from a line and a half to two lines in diameter, and filled nearly to a level with the surface, with large whitish granulations, surrounded by a sero-purulent secretion. In two or three smaller ones, this matter has dried into a scab, completely filling them up, while in one or two others, which are very deep, but retain their original breadth, no granulations are to be seen, but they appear filled with serum. In the side, the wound which penetrated the lower part of the mamma, is scabbed over. Most of the others are filled with the remains of the sloughs, and hardened pus. They are each surrounded by a distinct areola. She still takes the Tart. Ant. regularly. Same prescription and dressings.

Sept. 4th. Face slightly coloured. Pulse full, 135 to 140. Did not sleep well during the night, owing to a *diarrhœa,* and a very severe pain extending from right side across the epigastric region, which came on yesterday afternoon. Some of the wounds in the side appear healed, and all the orifices, both in side and arm, are filled with florid granulations. Tart. Ant. discontinued. Twenty drops of laudanum ordered, and blister to right side.

Sept. 5th. Face natural. Slept well. Pulse full, 130. Pain in side entirely removed by blister. Same general state. Wounds not examined.

Sept. 6th. Complained last night of severe pain in lower part of back, where there exists no redness of skin. Pulse full, 125 to 130. Not quite so strong in the injured arm as in the opposite one.

Face slightly colored, free from anxiety. *Diarrhœa* continues. Complains of no pain in any part at present. The wounds in the arm do not appear to have altered perceptibly for two or three days. Those in the side, which were all covered by the blister, are rather large, with whitish edges, and filled with a whitish pus. The skin is no longer tense around those of the arm or side, and those in the arm are less deep. Complained at 8 o'clock last night of great sensation of cold in the wounded arm and in the breast, but the skin offered to the feel a temperature greater than natural. Laudanum and effervescing mixture.

Sept. 7th. No perceptible change. Slept well. No pains since the 5th. Has had no return of chilliness.

Sept. 8th. Orifices in side, which were covered by blister, slightly painful. Most of the wounds in the arm are healed; the rest filled with a thick yellow pus. Same general state. Free from pain. Cheek slightly flushed.

Sept. 10. Same general state; no pain in any part; has had no return of chill since the 1st; orifices in the side filled with concrete pus; those of arm filled with red, healthy granulations, and pressure produced a discharge of clear yellow pus.

Sept. 13. At 11½ o'clock A. M., *a very severe chill*, which lasted an hour and a half, and was followed immediately by *profuse perspiration*, which continned for upwards of twelve hours, without pain.

Sept. 14. Skin cool; pulse moderate; countenance pale, free from anxiety. Patient feels well, and suffers no pain; very slight change in appearance of wounds. Since the 10th, they are slowly healing.

Sept. 15. Free from pain; countenance natural; pulse 96, moderately full; at half past eleven o'clock yesterday a *sense of chilliness* came on, which alternated with profuse perspirations, without the intervention of a hot stage, till the evening. During the night she slept well; all the wounds are rapidly cicatrizing; a small shot was taken from an ulcerated spot in the back. Ordered one grain of sulphate of quinine every hour from eight till eleven to-day.

Sept. 16. Same general state; free from pain in any part; took the quinine yesterday as directed. *Chill* (not amounting to a shake,) came on yesterday at half past three, continued for about two hours, and was immediately followed by perspiration. Slept well during the night; wounds rapidly cicatrising; quinine to be continued, one grain every hour, until the appearance of a chill.

Sept. 17. *Sense of chilliness* came on yesterday at half past twelve A. M. The patient did not shake;—had taken five quinine pills. The chill lasted between two and three hours, and was followed immediately by moderate perspiration. Slept well. Wounds of side entirely cicatrised. Those of arm nearly so. The abrasion of skin in the back, caused by the oblique passage of shot, has given rise to three ulcerations about two inches in length, and two lines in breadth, which are now inflamed, owing probably to the patient's resting upon that part. Poultice to these wounds; ordered to lie upon left side. Quinine continued, with a moderate use of port wine, and a free diet.

Sept. 18. Took the quinine yesterday, and had no return of chill. Slept well. This morning the cheeks slightly coloured; skin warm; pulse small, about ninety-six; mouth open; lips and teeth slightly coated with sordes. The wounds in arm are healing rapidly; the tongue whitish. The mouth feels very dry; great thirst. The ulcerations in the back are less inflamed and less painful. Bowels free.

Sept. 19. Face thin; slightly anxious. Slept well. Complains of no pain in any part. Took four grains of quinine yesterday morning. and had no sensation of chilliness. Wounds in side entirely cicatrised; those of the arm almost entirely so. There is one orifice directly opposite the centre of the external condyle of the right arm, which appears cicatrised; but upon pressing the arm immediately above it a profuse, watery, yellowish discharge issues from it; very fetid, and possessing the odour of carious bone. On examining more minutely, a small hole about the size of a pin's head is found in the centre of the cicatrix, and a probe introduced passes for a quarter of an inch into the substance of the bone, which feels ragged.

Sept. 21. Yesterday afternoon commenced expectorating yellowish sputa, *resembling those of pneumonia, but more yellow.* Slightly viscid. The amount of expectoration since last evening is about two ounces. To day it consists of fluid mucous, coloured by bile; very diffluent, and possessing no viscidity. The clothing of the bed on which she has wiped her lips, is also tinged in several places by bile. Countenance thin, slightly anxious. Has had no pain in the side. Pulse tolerably full, 124. Respiration easy.

Sept. 22. Cheeks and nose flushed and red. Pulse full, depressible, 136. Respiration 32, slightly laborious. Has expectorated since yesterday morning about an ounce and a half of the same bilious sputa. The tongue and lips are tinged of a dark yellow. No icterus. She has had no pain in the side, of which the wounds are perfectly healed. The only orifice in the arm which remains unhealed is opposite the centre of the external condyle, and gives issue to a moderate quantity of watery and extremely fœtid discharge. A sound introduced passes a quarter of an inch into the thickness of the bone. She breathes with a little difficulty, and, at each breath, a slight rattling is heard in the throat and upon applying the ear to the posterior part of right side of the chest, the "bruit de gargouillement" is very marked over the whole extent. Very restless during last night, although complaining of no pains. She has been daily becoming emaciated since her entrance. Ordered generous diet.

Sept. 23. Same general state. Suffers no pain. Countenance emaciated; pale in general, but coloured over the malar bones, and over the bridge of the nose. Expectoration since last evening about two ounces of the same fluid as hitherto. Pulse tolerably full, 128. Respiration, 40. Speaks in a husky, and sometimes in a squeaking voice, owing to the collection of mucous and bile in her throat.

Sept. 24. General health improved lately. She walks about the chamber. Appearance of wounds unchanged. Expectorated since yesterday about two ounces of mucous and bile. Suffers no pain. Pulse full, 135. Respiration, 40.

Sept. 25. Countenance improved. Slept well, except when awakened by the cough, which was very frequent. Has expectorated since yesterday at noon about six ounces of the same fluid as before. Mouth tinged of a yellow colour; sputa very bitter to her taste. Suffers no pain in any part; the wound in the external condyle still discharges a watery, fœtid fluid.

Sept. 26. Cheeks more full; countenance good and free from anxiety. Expectoration of about six ounces since yesterday morning. No pain.

Sept. 27. Same general state; countenance natural. Strength increasing continually. Expectoration of about six ounces since yesterday noon—the expectoration is, however, of a much lighter colour, and contains some portions floating on the surface which are almost free from colour. She complains still of a very bitter taste in the mouth whenever she expectorates.

Sept. 28. General health continues rapidly improving. The expectoration has not diminished in quantity, but is losing its deep bilious colour. The orifice opposite the external condyle is still open, and discharges a watery, fœtid matter.

Sept. 29. The patient is sitting up and dressed. Countenance, smiling. Says she feels perfectly free from pain and quite strong. The expectoration during yesterday amounted to about four ounces; but, during the night, and up to the present moment, although she has coughed a great deal, she has not expectorated. Her voice is much stronger and quite natural.

Oct. 4. Continual increase of strength since the last date. The expectoration has been constantly diminishing in quantity, and is at present scarcely coloured by the bile, and much less bitter. The orifice opposite external condyle is nearly closed.

Oct. 12. Continual increase of strength. Walks about her chamber without pain. Appetite excellent. Expectoration very moderate, consisting chiefly of saliva. The patient is entirely free from pain. The wounds are healed, and covered by sound skin. The motions of the elbow joint are unimpaired. The pulse little less feeble than on the sound side. I find, however, that she has not possessed the entire use of her hand since the moment of the accident. The fingers are demiflexed upon the hand, and the hand demiflexed upon the forearm. She can close the fingers completely, but is unable perfectly to extend them; and when the limb is supine, she can flex the wrist, and place the hand in a state of pronation. When, on the contrary, the hand is prone, she can neither extend the wrist nor supinate the forearm.

The radial nerve has clearly been paralyzed in its course along, or before entering, the humeral gutter, by one or more of the shot; for its branches to the triceps are unimpaired in their influence; and it was probably from the profunda, and not from the brachial artery, that the hæmorrhage originated. The return of pulse to the wrist corroborates this natural supposition.

Further remarks upon this case are intentionally deferred until the completion of this series of cases.

